# Testing projected wild bee distributions in agricultural habitats: predictive power depends on species traits and habitat type

**DOI:** 10.1002/ece3.1579

**Published:** 2015-09-23

**Authors:** Leon Marshall, Luísa G. Carvalheiro, Jesús Aguirre‐Gutiérrez, Merijn Bos, G. Arjen de Groot, David Kleijn, Simon G. Potts, Menno Reemer, Stuart Roberts, Jeroen Scheper, Jacobus C. Biesmeijer

**Affiliations:** ^1^ Naturalis Biodiversity CenterLeidenThe Netherlands; ^2^ Department of GeographyUniversity of NamurNamurBelgium; ^3^ Institute of Integrative and Comparative BiologyUniversity of LeedsLeedsUnited Kingdom; ^4^ Institute for Biodiversity and Ecosystems Dynamics (IBED)University of AmsterdamAmsterdamThe Netherlands; ^5^ Louis Bolk InstituutDriebergenThe Netherlands; ^6^ Alterra – Wageningen URWageningenThe Netherlands; ^7^ Resource Ecology GroupWageningen UniversityWageningenThe Netherlands; ^8^ Centre for Agri‐Environmental ResearchSchool of Agriculture, Policy and DevelopmentUniversity of ReadingReadingUnited Kingdom; ^9^ European Invertebrate Survey Kenniscentrum Insecten – The NetherlandsLeidenThe Netherlands; ^10^ Departamento de EcologiaInstituto de Ciências BiológicasUniversidade de BrasíliaBrasília70910‐900Brasil

**Keywords:** Arable fields, MAXENT, model validation, orchards, traits, wild bees

## Abstract

Species distribution models (SDM) are increasingly used to understand the factors that regulate variation in biodiversity patterns and to help plan conservation strategies. However, these models are rarely validated with independently collected data and it is unclear whether SDM performance is maintained across distinct habitats and for species with different functional traits. Highly mobile species, such as bees, can be particularly challenging to model. Here, we use independent sets of occurrence data collected systematically in several agricultural habitats to test how the predictive performance of SDMs for wild bee species depends on species traits, habitat type, and sampling technique. We used a species distribution modeling approach parametrized for the Netherlands, with presence records from 1990 to 2010 for 193 Dutch wild bees. For each species, we built a Maxent model based on 13 climate and landscape variables. We tested the predictive performance of the SDMs with independent datasets collected from orchards and arable fields across the Netherlands from 2010 to 2013, using transect surveys or pan traps. Model predictive performance depended on species traits and habitat type. Occurrence of bee species specialized in habitat and diet was better predicted than generalist bees. Predictions of habitat suitability were also more precise for habitats that are temporally more stable (orchards) than for habitats that suffer regular alterations (arable), particularly for small, solitary bees. As a conservation tool, SDMs are best suited to modeling rarer, specialist species than more generalist and will work best in long‐term stable habitats. The variability of complex, short‐term habitats is difficult to capture in such models and historical land use generally has low thematic resolution. To improve SDMs’ usefulness, models require explanatory variables and collection data that include detailed landscape characteristics, for example, variability of crops and flower availability. Additionally, testing SDMs with field surveys should involve multiple collection techniques.

## Introduction

Pollinators are responsible for the pollination of over 80% of flowering plants (Ollerton et al. 2011), and the vast majority of global food crops benefit from animal pollination, with approximately half of these crops being highly dependent (Klein et al. 2007). While the honeybee (*Apis mellifera* L.) is considered the most economically valuable pollinator species for agriculture, wild pollinators can be more efficient per individual in enhancing the yield and quality of many crops (Klein et al. 2007; Garibaldi et al. 2013). Yet, their diversity has declined in Europe (Biesmeijer et al. 2006; Dupont et al. 2011; Bommarco et al. 2011; Carvalheiro et al. 2013) and elsewhere (Bartomeus et al. 2013; Martins et al. 2013). These declines have been attributed to a multitude of factors, such as land‐use intensification, climate change, alien species, and pests and pathogens (Potts et al. 2010; Vanbergen and The Insect Pollinators Initiative 2013). Several pollinator‐friendly practices have been, and continue to be, applied to provide seminatural and natural resources within agricultural landscapes (Kleijn et al. 2011; Garibaldi et al. 2014). However, as wild pollinators often require specific environmental conditions (Cane et al. 2006), the efficiency of such practices can depend on the characteristics of the surrounding landscape and other environmental variables (Scheper et al. 2013). Understanding which environmental factors determine where wild bees occur in the landscape is essential for the success of such targeted interventions.

Species distribution models (SDMs) can help in understanding how the distribution of and decline in wild bee species is regulated by land‐use and climate variables (Elith and Leathwick 2009). Due to the increase in computer power and data availability, species distribution modeling is becoming a widely used ecological tool in studies of biodiversity, predicting occurrence of species in unknown areas, and predicting future occurrences (Franklin 2013). These predictions can help prioritize areas in need of conservation interventions and estimate the impact of environmental change, such as human land‐use changes (Guisan and Thuiller 2005; Polce et al. 2013). However, while SDMs are generally based on haphazardly collected data of varying spatial and temporal scale (e.g., museum collection data) and aggregated over a number of years, they are often used to test hypotheses at finer scales and at particular moments in time (Guisan and Thuiller 2005). The efficacy of SDMs for these purposes is therefore a reason of concern.

The importance of testing the accuracy of SDMs is widely recognized (Elith and Leathwick 2009). However, such accuracy tests often use subsets of the same collection data used to build the model. These tests violate the independence expected between training and testing data (Bahn and McGill 2013). Additionally, these tests require a large number of collection points for the data partitioning to be valid (Allouche et al. 2006; Fawcett 2006). Testing the models by collecting independent presence data is the ideal approach, but is rarely applied due to logistic constraints, particularly when dealing with highly mobile organisms (Evangelista et al. 2008; Peltzer et al. 2008). Therefore, for many animal species, it is uncertain whether SDMs can accurately predict species presence in specific locations, and hence, how useful and reliable the results can be in guiding policy for the protection of biodiversity, or estimating the presence of economically valuable species.

In this study, we test the performance of SDMs in correctly predicting wild bee occurrences from recent field surveys and how this varies between species and landscape. As the effects of disturbance and fragmentation depend on sociality, body size, and nesting behavior of bees (Bommarco et al. 2010; Williams et al. 2010; Brittain and Potts 2011), we expect the performance of the SDMs to depend on these traits. Previous studies show that specialized, plant and amphibian species, with specific habitat requirements, are more accurately modeled (Evangelista et al. 2008; Peltzer et al. 2008; Newbold et al. 2010), and we hypothesize that the bees specialized in habitat and feeding will have higher habitat suitability predictions for their occurrences than generalist, widespread species. Additionally, we expect that rarer species will have higher predicted habitat suitability due to the reduced geographical range they usually occupy (Franklin et al. 2009; Rebelo and Jones 2010). Finally, as the SDMs will be based on species records with variable spatial and temporal precision, we hypothesize model predictions in agricultural habitats which have a greater temporal stability (e.g., orchards) will have higher suitability values than for agricultural areas subjected to accentuated temporal changes (such as crop rotation) or subjected to ephemeral establishment of areas rich in flower resources (e.g., wildflower strips).

## Methods

### Species distribution model development

This study focuses on the Netherlands, a region for which we have access to relatively extensive and detailed data on species distributions, land use, and climate. The bee collection data were provided by European Invertebrate Survey (Peeters et al. 2012). We used records collected since 1990, and due to the number of available explanatory variables, we included species for which we had more than 30 recorded observations. This led to a total of 193 species across 25 genera (from a total availability of 304 species in 30 genera). A total of 43 989 observations were used to model the species’ distributions. The number of collection points per species modeled ranged from 31 (*Bombus cryptarum* Fabricius, *Lasioglossum pallens* Brullé, and *L. rufitarse* Zetterstedt) to 1862 (*B. pascuorum* Scopoli).

We modeled the distribution of these 193 species across the Netherlands using R (R Core Team, 2012) with package biomod2 (Thuiller et al. 2009) and the species distribution modeling algorithm Maxent (Phillips and Dudík 2008). We chose Maxent because it has previously performed well on similar data for a variety of evaluation measures and is robust against overfitting (Phillips et al. 2006; Aguirre‐Gutiérrez et al. 2013). The models were constructed with the BIOCLIM climate variables obtained from WORLDCLIM database (Hijmans et al. 2005), and land‐use variables obtained from the Dutch rural land‐use file version six (Hazeu et al. 2012) and the TOP10NL (Kadaster, 2012). The original resolution of the land‐use variables was 25 × 25 m; to match the coarser resolution of the bee collections and climate data, we rescaled the land‐use data to 1 km² by calculating the percentage cover (i.e., percentage of 25 × 25 m cells) of each land‐use class within each 1 km^2^.

Some precipitation and temperature variables for different parts of the year (i.e., warmest, coldest, and wettest quarters of the year) were strongly correlated (Pearson's pair‐wise correlation coefficient >0.7). In these situations, we selected the variable thought to have a greater impact on the distribution of bees, such as the variables related to the periods when bees are most active, for example, the warmest quarter. To minimize the overall number of explanatory variables in the model and avoid problems of overfitting, we ran initial MAXENT models for each species with all environmental variables available (27 variables) and then looked at the variable importance value of each variable across all species. We then selected the variables that were consistently among the three most important variables for each species and removed those that were not. The final SDM incorporated thirteen variables: seven land‐use variables, five climate variables, and elevation (see Table S1).

Maxent requires a background sample to be selected from the covariates included in the model (Elith et al., 2011; Phillips et al. 2009). We used target‐group sampling to select our background points (Phillips et al. 2009; Mateo et al. 2010). We specified that this background sample could only be selected from areas where wild bee species have been found since 1990. This approach is more objective and realistic than taking the background sample from sites that have not been sampled, accounting for potential sampling bias (Phillips et al. 2009; Elith et al. 2011), and provides more accurate results (Mateo et al. 2010). We ran the model 11 times for each species: 10 times with random subsets of 80% of the data and once with 100% of the data. Using a common procedure of validation of SDMs, we then used the remaining 20% of the data to produce area under the curve (AUC) values, which is a measure of the proportion of instances correctly predicted against the proportion of absences incorrectly predicted as presences (Jiménez‐Valverde 2012). All species models had an AUC of at least 0.6.

We validated the full models (run with 100% of the data) with independent datasets collected during field surveys (see methods below). Model output consisted of a habitat suitability score between 0 and 1 for each species per 1 km^2^, with 0 indicating not suitable and 1 most suitable.

### Field surveys

The data used to test the predictive performance of the SDMs were collected from four independent studies, details of which are described below (for site locations see Figure S1). Bee species collected and identified to species level were used to test the models. The different studies were independent of each other, data being gathered in different time periods, by different collectors, and using a systematic survey across several sites and over short time periods. They were experimentally set‐up to test particular research questions associated with specific farm types and habitats: arable oilseed rape fields and associated field margins; arable fields with wildflower strips, and apple and pear orchards. While these agricultural landscapes do not represent Dutch farmland as a whole, they cover important types of agricultural landscape with different levels of temporal stability. Orchards are perennial crops maintained for several years; arable fields have annual crops, with crop species rotating every 1 or 2 years. Measures to enhance biodiversity in arable fields (permanent field margins vs. annual wildflower strips) will also interfere with the temporal stability of the landscape. The studies also differed with respect to the sampling methods used.

Furthermore, the SDMs presented here are independently validated based on data from agricultural sites only. In order to fully understand the efficacy of SDMs for modeling wild bee species distributions, natural habitats can also be included, in which bee diversity is much larger than in agricultural habitats (Ricketts et al. 2008).

#### Arable oilseed rape fields and field margins (sampling method: Transect)

Data were collected in 2011 and 2012 in 16 arable oil seed rape fields and surrounding boundaries located in the eastern part of the Netherlands. Bee surveys were conducted along 150 m^2^ transects (15 min pure collecting time per transect). When sampling within fields, two transects of 1 m × 150 m were used, one located at the edge of the field and one located in the center of the field. Field boundary transects varied in size depending on the length and width of the field boundaries (but were in most cases 2 m × 75 m). Oil seed rape fields were surveyed twice a year during oil seed rape flowering, and the field boundaries were surveyed four times a year: twice during and twice after the flowering period of the oil seed rape. Bees were collected using net and hand trapping and identified to species level in the laboratory.

#### Arable fields with wildflower strips (sampling method: Pan Trap)

In 2011 (first season of wildflower strips) and 2012 (second season), data were collected on 68 arable fields throughout the Netherlands using pan traps. Wildflower strips had been established along the edge of each arable field. Each wildflower strip was 3–9 m in length. The arable fields consisted of potato, sugar beet, or cereal crops. Pan trapping was conducted once at each site. All pan traps were yellow and four were placed at each site, in a square formation two traps in the wildflower strip and two traps in the field each 20 m apart. Each set of pan traps was left for a 24‐h period. All species of insects collected in the pan traps were identified, the majority to species level.

#### Apple and pear orchards (sampling method: Transect)

Six apple and six pear orchard locations were sampled in 2010 and 2011, and 15 apple orchards were sampled in 2013. All sites were located more than 3 km apart within the province of Gelderland in the Netherlands. Flower visiting bees were surveyed using transect walks. Each orchard was surveyed twice per year during blooming, once in the morning and once in the afternoon with at least three and at most 7 days separating surveys. In each orchard, bees were surveyed using a single transect between two rows of trees along the length of each orchard with the transect subdivided into 25‐m‐long plots (mean number of plots per orchard ± SE: 8.5 ± 1.0 for apple in 2011 and 2012; 9.7 ± 0.5 for pear in 2011 and 2012; exactly 12 for apple in 2013). Each transect plot was surveyed during a 10‐minute period. All flower visitors were collected by net and hand trapping. Easily recognizable species were generally identified in the field; all other species were collected and identified in the laboratory.

#### Apple Orchards (sampling method: Pan Trap)

In 2013, field surveys were performed at nine apple orchards throughout the Netherlands. Field surveys of bee diversity were conducted using pan traps (Westphal et al. 2008). Each farm was located within a 1 km² square landscape sector that corresponded to the scale and positioning of our SDM. Pan trapping was conducted on three separate occasions: before, during, and after apple flowering. For each 1 km² site, eight pan traps were positioned, four within the Elstar cultivar (one at each corner) and four located outside the orchard but within the 1 km² zone. Each pan trap set consisted of three pan traps (yellow, blue, and white) and was left for a period of 24‐h. Bees present in the pan traps were separated from other insect groups and identified to species level.

### Testing the model with independent datasets

In this project, the performance of the SDM is assessed as the habitat suitability (0–1) provided by the SDM for the areas where individual wild bees were collected during independent surveys. Suitability values can be considered as a percentage of chance that a species will be present in the area (see the interpretation of Elith et al. (2011) of the MAXENT logistic output). Therefore, we consider the SDMs with higher habitat suitability values for collected occurrences to have superior predictive performance. Furthermore, the habitat suitability value contains more information than the usual binary (presence or absence) classifications based on specificity and sensitivity calculated statistics (Bahn and McGill 2013). We analyzed the predictive performance of the SDMs only for species that were collected during the independent field surveys. We did not analyze predictive performance for species not found during the field surveys as we cannot assume that that absence during the survey is indicative of true absence from the site.

**Figure 1:**
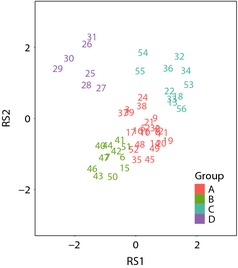
Results of Hill & Smith multivariate approach based on six biological traits across 2 axes, (RS1 and RS2). Four groups selected. Groups A‐D (See Table 1). RS1 is positively directed by oligolectic, solitary, below ground bees. RS1 is negatively directed by social, habitat generalist aboveground bees with long flight periods. RS2 is positively directed by large, oligolectic, social bees which nest aboveground. RS2 is negatively directed by polylectic below ground nesting bees (see Table S4). Each number refers to a bee species listed in alphabetical order (see Table S2).

To test whether the predictive performance of SDMs depended on species traits, we divided the 56 noncleptoparasite species collected in our field studies into trait groups (52 species were included in the final analysis; we removed four species, which were found only in forest edges near oil seed rape fields and not in either orchards or arable fields [See Table S2]). We considered six ecological traits from the “European bee traits database” (established by ALARM, www.alarm-project.ufz.de, and developed by STEP, www.STEP-project.net): habitat specialization, (continuous scale from 1 to 8 related to the number of habitat types a species occurs in, specialist to generalist), feeding specialization (oligolectic, feeding on one plant species or polylectic, feeding on multiple plant species), body size (intertegular distance of females, where the wings join the thorax), sociality (solitary or social; social species included eusocial as well as primitively eusocial species, all others were classified as solitary), nesting habit (above or belowground, belowground species included any renters or excavators which used nests in the ground all others were considered aboveground), and length of flight period (period active during the year; from 8 to 36 weeks). We identified trait groups using the Redundant Hill & Smith dimensional scaling technique. This method was chosen as it allows for concurrent analysis of both categorical and continuous ecological trait data by defining the categorical variables by the means of the continuous variables (Hill and Smith 1976; Barnagaud et al. 2014). The analysis was conducted using R package ade‐4, which first uses principal component analysis to process the continuous variables and correspondence analysis for the categorical variables and then the Hill and Smith analysis to compare the relationship between the two (Dray and Dufour 2007). Four distinct species groups were selected (groups A–D; see Table 1; Fig. 1). The three most important variables involved in the analysis were nesting habit, feeding specialization, and sociality. Each group contained at least 5 species (See Table S2). We can typify group A as polylectic, habitat specialists; group B as small, polylectic, habitat generalists; group C as oligolectic, habitat specialists; and group D as large, polylectic, habitat generalists (consisting of *Bombus* species only). Two species were not clearly allocated to one of the above four groups *Megachile ligniseca* (Kirby) and *M. versicolor* (Smith, F.). However, they were classified as part of group C, with whom they share the most traits (Fig. 1).

**Table 1:** Trait summary of the four bee species groups selected using the Hill and Smith method of multiple correspondence analysis (MCA), based on six biological traits across 2 axis

Group	Habitat specialization	Diet specialization	Body size	Sociality	Nesting habit	Flight period	Dominant genera
A (26) – Small intermediate specialists	Specialists	Polylectic	Small	Solitary	Below	Short	Andrena
B (12) – Small generalists	Generalists	Polylectic	Small	Mixed	Below	Long	Lasioglossum
C (11) – Highly specialized bees	Specialists	Oligolectic	Intermediate	Solitary	Mixed	Short	N/A
D (7) – Large generalists	Generalists	Polylectic	Large	Social	Mixed	Long	Bombus

Numbers in brackets refer to the number of species selected in each group. Habitat specialization, continuous variable, representing the number of habitat types, from 1 (specialist) to 8 generalist. Diet specialization, factor oligolectic or polylectic (oligolectic, feeding on one plant species or polylectic, feeding on multiple plant species). Body size, continuous, intertegular distance of females (mm), sociality, factor, solitary or social. Nesting habit, factor, below, or aboveground. Flight period continuous, 4–36 weeks. Dominant genera, the genera that makes ≥70% of the species diversity in that group.

We tested whether the habitat suitability predicted by our SDMs for these 52 species varied between trait group (A–D) and habitat (orchard or arable field), using linear mixed effect models (LMM), with R package lme4 (Bates et al. 2013). The sampling method (transect vs. pan traps) used in the field surveys was also included as an explanatory variable in the LMM, to account for any possible methodological bias. Due to the nested structure of the data, multiple collection sites within separate studies, we included site within study as a random effect variable. Additionally, as the species collected were only a subset of all the species modeled for the Netherlands, we included species as a random effect variable.

Detailed collections of multiple individuals in the same area are required to predict the distribution of species abundance alongside habitat suitability predictions (Van Couwenberghe et al. 2013). Because of its scope and resolution, this was not feasible for our SDM. Nevertheless, we included the number of records used to build SDMs in the analysis as a proxy for species rarity and probability of detection.

We compared all possible combinations of the variables described above, and their two‐way interactions, and selected the most parsimonious model based on the lowest Akaike information criterion, corrected for finite sample size (AICc). We also compared the mixed effect models with the Bayesian information criterion (BIC), which punishes extra terms more harshly than the AIC and AICc (Burnham and Anderson 2002).

## Results

### Testing the model with independent datasets

A total of 446 individuals of 52 species (excluding cleptoparasites) were collected at 133 sampling locations and were used to analyze the predictive performance of our SDMs. The abundance and richness of wild bees varied between habitat types, species trait groups, and sampling technique (see Figures S2 and S3).

**Figure 2:**
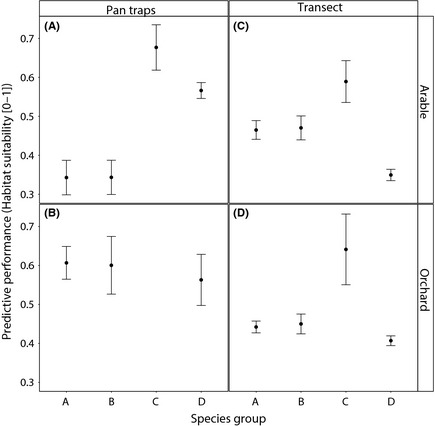
Mean and standard error of habitat suitability for collection points of the four species groups, in both landscape types (Orchard and Arable) and for both sampling techniques (Pan Trap and Transect). Group A = small, intermediate specialists, group B = small generalists, group C = highly specialized bees, group D = large generalist bees. See Table S3 for pairwise comparisons between effects.

The habitat suitability values obtained from the SDMs, for each of the occurrences collected, varied between the different types of habitat where the collection took place, and also among the different species trait groups (Table 2, Fig. 2). Although the number of records differed significantly between groups (see Figure S4), the habitat suitability of the model was not significantly affected by this variable (ANOVA, chi‐square test, *P* = 0.13). The sampling method used to collect the independent wild bee occurrences significantly affected the measure of SDM habitat suitability overall. Moreover, significant interactions were found between sampling and group and sampling and habitat type; the effect of habitat type decreased for transect collections and the effect of species trait groups was also lower for transect collections than pan trap collections (see Table 2).

**Table 2:** Effect of species trait group (G), sampling technique (S), and landscape type (L) on species distribution model predictive performance (habitat suitability of species occurrences). Number of observations was 436 of 52 unique species. *P*‐values were obtained from likelihood ratio tests where deviance between models with the term and without the term where compared. n.s = *P* > 0.05. The symbol “–” represents a variable not included in the model. All interactions where tested and those which contributed significantly to any of the models remained. Random terms (all models): “1 ¦ Study/Site,” “1 ¦ Species”

Response Variable	G	S	L	G:S	G:L	S:L	DF	AICc	∆AICc
Accuracy									
Model 1 (Best Model)	0.042	<0.001	0.1	<0.001	–	0.025	422	5636.1	0.0
Model 2	0.042	<0.001	0.1	<0.001	0.3	0.035	419	5638.9	2.79
Model 3	0.044	<0.001	0.1	<0.001	–	–	423	5639.0	2.9
Model 4	0.05	0.001	–	<0.001	–	–	424	5639.5	3.39
Null Model	–	–	–	–	–	–	431	5685.8	49.64

								BIC	∆BIC
Model 1 (Best Model)	0.05	0.001	–	<0.001	–	–	424	5687.7	0.0
Model 2	0.044	<0.001	0.1	<0.001	–	–	423	5691.2	3.47
Model 3	0.042	<0.001	0.1	<0.001	–	0.025	422	5692.2	4.51
Model 4	–	0.001	–	–	–	–	430	5701.4	13.71
Null Model	–	–	–	–	–	–	431	5706.0	18.31

Data were available for all groups in each of the habitat types and collection techniques except group C. Species of this group were not collected in pan traps within orchards (Fig. 2B). Overall, the occurrences of highly specialized bees (group C) had higher average suitability values than the other three groups (Fig. 2); significantly more than group A and group B species (*P* < 0.036 and 0.037, Fig. 2, See Table S3). Furthermore, the modeled habitat suitability values for species occurrences from group D were significantly lower when comparing transects with pan traps (*P* < 0.001, See Table S3).

Overall the bee species collected in orchard habitats had higher predicted habitat suitability than those collected in arable field habitats (Table S3). This result was particularly accentuated for bees collected with pan traps (Fig. 2A and B). Furthermore, within orchard sites, the pan trap collected bees were more accurately predicted than the transect‐collected bees (Fig. 2B and D).

## Discussion

Field surveys are rarely used to test species distribution models (SDM), particular those investigating spatial patterns of highly mobile animals such as bees (Fielding and Bell 1997; Jiménez‐Valverde et al. 2008). We analyzed the SDM habitat suitability scores of independent wild bee occurrences, and we show that the performance of SDMs to predict wild bee occurrences in field surveys depends on species traits and on the characteristics of the target habitat and sampling technique. Below we discuss the implications of these findings and the limitations of our study.

### Variation of model predictive performance among different species trait groups

Wild bee species with different traits can have contrasting responses to environmental conditions. Specialist bees have been shown to be more strongly affected by agricultural intensification, habitat loss, and fragmentation than generalists (Bommarco et al. 2010; Williams et al. 2010). Habitat and feeding specialists are generally more restricted in their range of suitable habitats, while large, generalist bees such as bumblebees have greater mobility and can meet their resource requirements in a wider range of habitats (Hanley et al. 2011). This probably explains the better model performance for highly specialized species, indicating that SDMs are better able to discriminate their more restricted habitats. Similar patterns have been demonstrated for other taxa (Evangelista et al. 2008; Peltzer et al. 2008; Newbold et al. 2010; Trumbo et al. 2011). This finding suggests that while the 1 km² resolution used in this study is appropriate for predicting the distribution of specialized bee species, a more detailed sampling data or different set of predictor variables would likely be needed to obtain better predictions for more generalist species. Furthermore, the differences between model predictive performance for specialized and generalist bees suggest that the SDM may be more useful for conservation purposes focused on more specialized species which are more likely to suffer declines (Biesmeijer et al. 2006), than for predicting crop pollinators which are commonly more generalist species (but see Polce et al. 2013).

Model performance varied between studies using different sampling techniques which suggests that pan trap and transect collections sample different parts of a bee community and that the SDMs do not predict these subsets equally. Indeed, Cane et al. (2000) found that transect walks sampled the bee community better than pan trapping, where many abundant and specialized bee species were absent. In contrast, Westphal et al. (2008) showed that pan trapping and transects sampled similar species composition, but that pan traps generally sampled more of the wild bee community than transect surveys. However, these results are strongly limited by the intensity of each method, the experience of the transect surveyors, whether the pan traps are painted UV bright and whether they were placed at vegetation height. Bumblebees (trait group D: large generalists) showed distinct trends related to sampling technique. The occurrences of bumblebees collected during transects had lower predicted habitat suitability in the models than those from pan traps. This difference was particularly marked in arable fields which were generally predicted in our SDM to be unsuitable habitats, but where bumblebees were frequently detected. Bumblebees can travel long distances and respond very rapidly to the presence of unexpected mass‐flowering events of attractive crops, such as when annual crops like oil seed rape start blooming (Hanley et al. 2011). However, bumblebees and other highly social species have been shown to have higher flower and site constancy than smaller, solitary bees (Osborne and Williams 2001; Gegear and Laverty 2004) and therefore may be less likely to be caught in pan traps. The use of multiple collection techniques for independently testing the performance SDMs is therefore essential (see also Westphal et al. 2008).

### Variation of model predictive performance among different landscapes

Overall, the wild bees collected in orchards were predicted with significantly higher suitability values than the species collected in arable fields, particularly when using pan traps and for small, mainly solitary bees (groups A and B). In this study, the category “arable fields” includes a variety of crops, some having periods of intense flowering very attractive to bees (e.g., oil seed rape, Delaplane and Mayer 2000), while others are less attractive to bees (e.g., sugar beet and wheat, Delaplane and Mayer 2000). Additionally, in annual crop fields, the type of crop is frequently rotated, and so continuously changes between years (Stoate et al. 2001), and several were subjected to recent changes as a result of agri‐environment schemes (AES) that involved the establishment of field margins or annual wildflower strips (Kleijn et al. 2006). These characteristics make arable fields far more temporally unstable than orchards. The species data used to build the SDMs spans 20 years and during that time it is likely that the arable fields have comprised a variety of crops and for the majority of this time AES had not been implemented. AES that increase flowering species within farmland (e.g., implementation of wildflower strips, establishing field margins) also increase the time window in which flower resources are available (e.g., Haaland et al. 2011) and provide temporary connectivity between less desirable habitat types, for a number of insects including bees (Carvalheiro et al. 2012; Holzschuh et al. 2013). The results suggest that the variables used to construct the SDMs do not represent the AES or the seasonal changes in crop flowering, which is reflected by the wild bee occurrences in otherwise predicted unsuitable habitats.

The high heterogeneity of this landscape type combined with a lack of spatial and temporal cover in the data used to build the SDMs is hence a likely explanation for the poorer performance of SDMs in arable fields in comparison with orchards. Again this reinforces the idea that SDMs of this type are less suitable for predicting pollination service delivery to arable crops than for predicting the occurrence of threatened species and their habitats.

### Implications for future studies using species distribution models

The analysis implies that the models with higher predictive performance have correctly represented the ecological niche of a species. SDMs are often used to make decisions regarding areas of conservation importance or also in the case of pollinators, where crops and pollinators overlap (Franklin 2013; Polce et al. 2013). Therefore, models with habitat suitability scores strongly correlated to temporally independent presences will have a higher efficacy in decision making. The results of our study suggest that studies using SDMs to predict bee species occurrences would benefit from more specific information about landscape type, crop type, including fine‐scale vegetation and AES data and information on flower availability within the landscape during different seasons of the year (sampling season) (Pearce et al. 2001). Unfortunately, such detailed information is rarely available, and the efficacy of long‐term collection data are limited by the historically available land‐use and climate information with which to model it. However, increased thematic resolution in the future, specifically for agricultural land use should assist in increasing the performance for certain species trait groups whose distributions are not accurately predicted by the lower thematic resolution of the current models. Temporally unstable habitats represent another difficulty for the development of valuable SDMs. Our results imply that a particular habitat is only suitable under certain conditions, such as when wildflower strips are blooming or when certain crops are flowering. As climatic and land‐use characteristics are subject to annual variation, and as pollinators can be susceptible to small scale habitat changes (e.g., presence of flower strips within farmland, Scheper et al. 2013), the model data are likely to be too coarse temporally to accurately predict the suitable habitat of a species at a specific moment in time. Species collection data, particularly those aggregated in museum collections generally cover long time periods, whereas crop rotation and AES occur in the short‐term. This suggests that temporal variation between habitat and species will remain difficult to separate in distribution models, and habitat suitability conclusions for fine‐scale landscape features will be difficult to produce. To overcome these caveats, SDMs need to be built with data specific to the year and season that a species was sampled. For example, in the Netherlands, AES are organized as regional collectives. Therefore, SDMs built and tested with detailed information from before and after the introduction of AES landscape features can be used to model the effectiveness and the changes resulting from AES and ensure ongoing monitoring and help determine future policy decisions.

Information on biotic interactions (e.g., bumblebee cleptoparasites and bumblebee hosts) can also increase the predictive performance of the wild bee SDMs (Giannini et al. 2013). This suggests that where clear ecological relationships are present including biotic information should improve the SDMs, particularly for the more generalist species which were not adequately modeled by climate and land use alone.

## Conclusions

Species distribution models are an important tool in ecological studies that can provide guidance for conservation management action and potentially also for management of ecosystem services. By comparing the predictions of SDMs developed for multiple bee species with independently collected field data, we show the performance of such models is highly dependent on species traits and on the spatial and temporal heterogeneity of the targeted habitat. While our analysis has only considered wild bees the results are not restricted to wild bees and suggest that other mobile and functionally varied species groups related to agricultural crops (e.g., hoverflies) may show similar trends to what we have observed here.

## Conflict of Interest

None declared.

## Supporting information


**Figure S1.** Field‐survey locations by landscape type and collection technique.

**Figure S2.** Diversity (number of species) collected at each site.

**Figure S3.** Abundance (number of individuals) collected at each site.

**Figure S4.** Average number of records per species group, with standard deviation error bars.

**Table S1.** List of Environmental Variables include in MAXENT species distribution modelling.
**Table S2.** List of Species per species trait group.
**Table S3.** Post hoc multiple pair wise comparison of difference in least square means, table for all significant interactions as selected in best model (AICc).
**Table S4.** Column coordinates for species traits used in group selection ordination analysis.
