# Supplementary material for: Testing projected wild bee distributions in agricultural habitats: predictive power depends on species traits and habitat type
Source: Ecol Evol. 2015 Sep 23;5(19):4426–36. doi: 10.1002/ece3.1579 (PMC4667819; doi:10.1002/ece3.1579)
Supplement: Supplementary file 5 — Table S1. List of Environmental Variables include in MAXENT species distribution modelling. Table S2. List of Species per species trait group. Table S3. Post hoc multiple pair wise comparison of difference in least square means, table for all significant interactions as selected in best model (AICc). Table S4. Column coordinates for species traits used in group selection ordination analysis. [file ECE3-5-4426-s005.docx]

**Supplementary Information**

**Tables**

**Table S1 List of Environmental Variables include in MAXENT species distribution modelling**

| **Type** | **Variables** |
| --- | --- |
| **Climate** | Mean Diurnal Range of Monthly Temperature |
|  | Mean Temperature of Warmest Quarter |
|  | Precipitation of Driest Month |
|  | Precipitation of Warmest Quarter |
|  | Temperature Seasonality |
| **Land Use** | Line Density of Simple Ditches |
|  | Line Density of Tree Alleys |
|  | Percentage Cover Agriculture |
|  | Percentage Cover Coniferous Forest |
|  | Percentage Cover Moors/Peats |
|  | Percentage Cover Sandy Soils |
|  | Percentage Cover Urban |
| **Topography** | Elevation |

**Table S2 List of Species per species trait group**

| **Species** | **Group** | **ID Number** | **Final Analysis** |
| --- | --- | --- | --- |
| *Andrena angustior* | A | 1 | YES |
| *Andrena barbilabris* | A | 2 | YES |
| *Andrena chrysosceles* | A | 4 | YES |
| *Andrena cineraria* | A | 5 | YES |
| *Andrena fucata* | A | 8 | YES |
| *Andrena fulva* | A | 9 | YES |
| *Andrena gravida* | A | 10 | YES |
| *Andrena haemorrhoa* | A | 11 | YES |
| *Andrena helvola* | A | 12 | YES |
| *Andrena humilis* | A | 13 | YES |
| *Andrena labiata* | A | 14 | YES |
| *Andrena nigroaenea* | A | 16 | YES |
| *Andrena praecox* | A | 18 | YES |
| *Andrena semilaevis* | A | 19 | NO |
| *Andrena subopaca* | A | 20 | YES |
| *Andrena tibialis* | A | 21 | YES |
| *Andrena vaga* | A | 22 | YES |
| *Andrena varians* | A | 23 | YES |
| *Colletes daviesanus* | A | 33 | YES |
| *Dasypoda hirtipes* | A | 34 | YES |
| *Lasioglossum sexstrigatum* | A | 49 | YES |
| *Panurgus calcaratus* | A | 56 | YES |
| *Andrena carantonica* | B | 3 | YES |
| *Andrena dorsata* | B | 6 | YES |
| *Andrena flavipes* | B | 7 | YES |
| *Andrena minutula* | B | 15 | YES |
| *Andrena nitida* | B | 17 | YES |
| *Halictus tumulorum* | B | 35 | YES |
| *Hylaeus communis* | B | 37 | YES |
| *Hylaeus confusus* | B | 38 | YES |
| *Hylaeus gibbus* | B | 39 | NO |
| *Lasioglossum calceatum* | B | 40 | YES |
| *Lasioglossum fratellum* | B | 41 | NO |
| *Lasioglossum leucopus* | B | 42 | YES |
| *Lasioglossum leucozonium* | B | 43 | YES |
| *Lasioglossum malachurum* | B | 44 | YES |
| *Lasioglossum minutissimum* | B | 45 | YES |
| *Lasioglossum morio* | B | 46 | YES |
| *Lasioglossum pauxillum* | B | 47 | YES |
| *Lasioglossum sexnotatum* | B | 48 | YES |
| *Lasioglossum villosulum* | B | 50 | YES |
| *Lasioglossum xanthopus* | B | 51 | YES |
| *Lasioglossum zonulum* | B | 52 | YES |
| *Anthophora plumipes* | C | 24 | YES |
| *Chelostoma florisomne* | C | 32 | YES |
| *Heriades truncorum* | C | 36 | YES |
| *Macropis europaea* | C | 53 | YES |
| *Megachile ligniseca* | C | 54 | NO |
| *Megachile versicolor* | C | 55 | YES |
| *Bombus hortorum* | D | 25 | YES |
| *Bombus hypnorum* | D | 26 | YES |
| *Bombus jonellus* | D | 27 | YES |
| *Bombus lapidarius* | D | 28 | YES |
| *Bombus pascuorum* | D | 29 | YES |
| *Bombus pratorum* | D | 30 | YES |
| *Bombus ruderarius* | D | 31 | YES |

**Table S3.** Post hoc multiple pair wise comparison of difference in least square means, table for all significant interactions as selected in best model (AICc). P-values adjusted by single-step method. (Suitability ~ Type3 * Sampling + Group * Sampling + (1 | Study/Site) + (1 | Species))

| **Species Trait Group** | **Estimate** | **Error** | **p-value** | **Sig.** |
| --- | --- | --- | --- | --- |
| A-B | 0.0173 | 0.04777 | 0.98 |  |
| A-C | -0.1539 | 0.05553 | 0.035 | * |
| A-D | 0.0078 | 0.05257 | 0.99 |  |
| B-C | -0.1712 | 0.06217 | 0.037 | * |
| B-D | -0.0095 | 0.05937 | 0.99 |  |
| C-D | 0.1617 | 0.0646 | 0.068 | . |
| **Habitat Type** |  |  |  |  |
| Arable-Orchard | -0.0818 | 0.02432 | 0.003 | ** |
| **Sampling Technique** |  |  |  |  |
| PanTraps-Transect | 0.0932 | 0.02763 | 0.002 | ** |
| **Group:Sampling** |  |  |  |  |
| PanTraps A - Transect A | 0.0334 | 0.03602 | 0.98 |  |
| PanTraps A - PanTraps B | 0.0412 | 0.05787 | 1 |  |
| PanTraps A - Transect B | 0.0267 | 0.05534 | 1 |  |
| PanTraps A - PanTraps C | -0.1868 | 0.08105 | 0.27 |  |
| PanTraps A - Transect C | -0.0877 | 0.06143 | 0.81 |  |
| PanTraps A - PanTraps D | -0.103 | 0.06053 | 0.64 |  |
| PanTraps A - Transect D | 0.1519 | 0.05957 | 0.16 |  |
| Transect A - PanTraps B | 0.0079 | 0.05535 | 1 |  |
| Transect A - Transect B | -0.0066 | 0.04772 | 1 |  |
| Transect A - PanTraps C | -0.2201 | 0.07829 | 0.09 | . |
| Transect A - Transect C | -0.121 | 0.05462 | 0.31 |  |
| Transect A - PanTraps D | -0.1363 | 0.05667 | 0.22 |  |
| Transect A - Transect D | 0.1185 | 0.05274 | 0.3 |  |
| PanTraps B - Transect B | -0.0145 | 0.03643 | 0.99 |  |
| PanTraps B - PanTraps C | -0.228 | 0.08562 | 0.13 |  |
| PanTraps B - Transect C | -0.1289 | 0.06748 | 0.5 |  |
| PanTraps B - PanTraps D | -0.1442 | 0.06611 | 0.33 |  |
| PanTraps B - Transect D | 0.1107 | 0.06582 | 0.66 |  |
| Transect B - PanTraps C | -0.2135 | 0.08352 | 0.16 |  |
| Transect B - Transect C | -0.1144 | 0.06125 | 0.53 |  |
| Transect B - PanTraps D | -0.1297 | 0.0635 | 0.42 |  |
| Transect B - Transect D | 0.1251 | 0.05951 | 0.37 |  |
| PanTraps C - Transect C | 0.0991 | 0.07632 | 0.87 |  |
| PanTraps C - PanTraps D | 0.0838 | 0.08367 | 0.96 |  |
| PanTraps C - Transect D | 0.3387 | 0.08603 | 0.003 | ** |
| Transect C - PanTraps D | -0.0153 | 0.06862 | 1 |  |
| Transect C - Transect D | 0.2396 | 0.06527 | 0.007 | ** |
| PanTraps D - Transect D | 0.2548 | 0.02918 | <0.001 | *** |
| **Sampling:Habitat** |  |  |  |  |
| Arable PanTraps - Orchard PanTraps | -0.1439 | 0.04137 | 0.006 | ** |
| Arable PanTraps - Arable Transect | 0.0311 | 0.02964 | 0.71 |  |
| Arable PanTraps - Orchard Transect | 0.0114 | 0.03033 | 0.98 |  |
| Orchard PanTraps - Arable Transect | 0.175 | 0.04231 | <0.001 | *** |
| Orchard PanTraps - Orchard Transect | 0.1553 | 0.0423 | 0.003 | ** |
| Arable Transect - Orchard Transect | -0.0197 | 0.02474 | 0.85 |  |

**Table S4. Column coordinates for species traits used in group selection ordination analysis**

| **Species Trait** | **RS1** | **RS2** |
| --- | --- | --- |
| Habitat Specialisation | -0.7315954 | -0.2495703 |
| Body size | -0.3839457 | 0.7020075 |
| Lecty (Oligolectic) | 1.354881 | 0.9099223 |
| Lecty (Polylectic) | -0.25945 | -0.1742404 |
| Sociality (Social) | -1.29095 | 0.3822533 |
| Sociality (Solitary) | 0.430316 | -0.1274178 |
| Nesting (Above) | -0.49199 | 1.3259499 |
| Nesting(Below) | 0.120265 | -0.3241211 |
| Length of flight period | -0.86646 | -0.2017732 |

**Figures**

**Figure S1** Field-survey locations by landscape type and collection technique

**Figure S2** Diversity (number of species) collected at each site.

**Figure S3** Abundance (number of individuals) collected at each site.

**Figure S4** Average number of records per species group, with standard deviation error bars. Numbers of records per group ranged from: A- 65 to 987; B- 56 to 955: C- 88 to 545: D- 172 to 1862.
